# Supplementary material for: Surface Microbiome Profiling of Dental Elevators Using Third‐Generation Sequencing: Implications for Infection Control in Dental Practice
Source: Microbiologyopen. 2025 Dec 8;14(6):e70178. doi: 10.1002/mbo3.70178 (PMC12685768; doi:10.1002/mbo3.70178)
Supplement: Supplementary file 1 — customer_backup. [file MBO3-14-e70178-s001.zip › customer_backup/customer_backup/function_analysis/BugBase/treat/otu_contributions/order/treat.order.Gram_Positive.pdf]

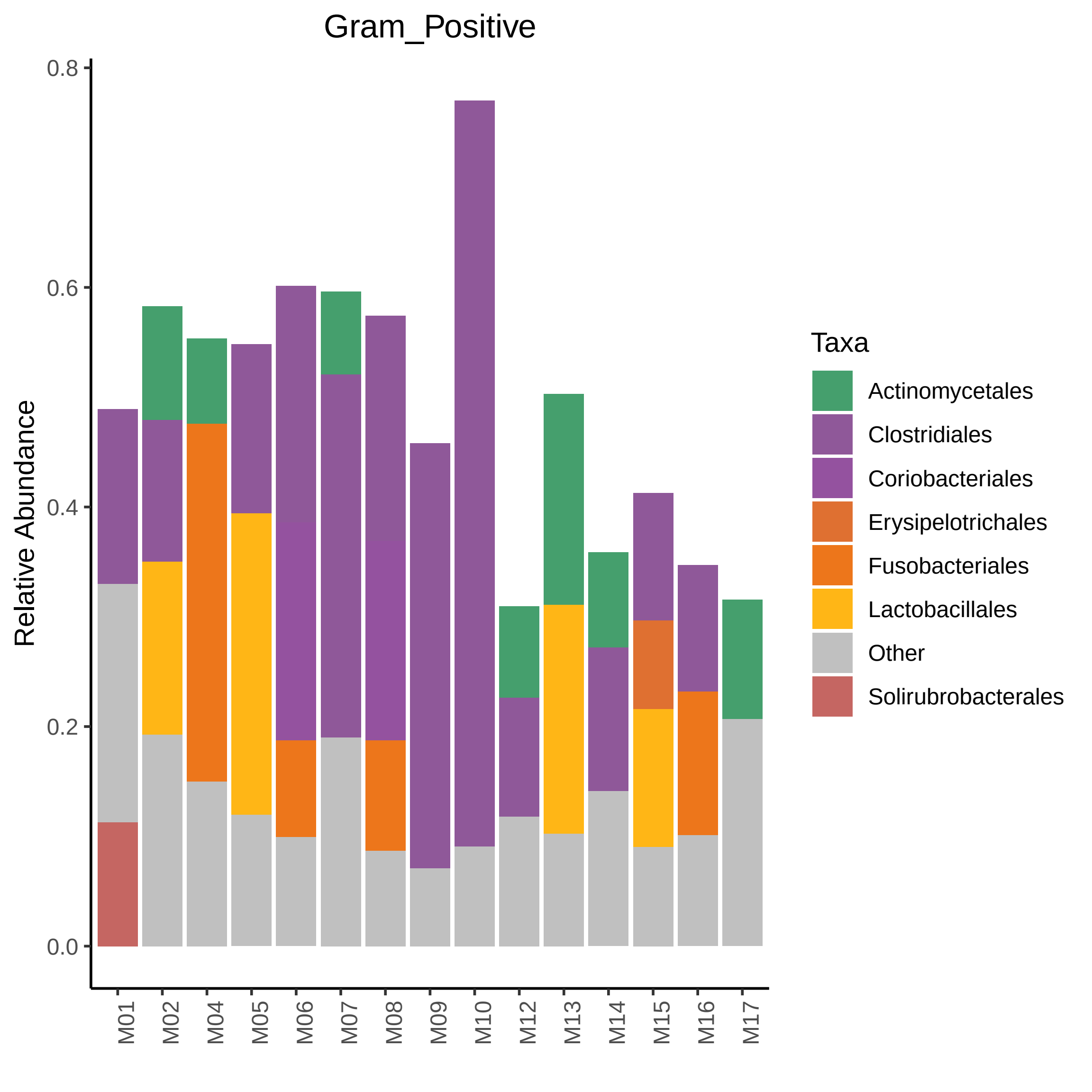

Gram_Positive
Relative Abundance
0.0
0.2
0.4
0.6
0.8
M01
M02
M04
M05
M06
M07
M08
M09
M10
M12
M13
M14
M15
M16
M17
Taxa
Actinomycetales
Clostridiales
Coriobacteriales
Erysipelotrichales
Fusobacteriales
Lactobacillales
Other
Solirubrobacterales
